# Slotted ALOHA Based Practical Byzantine Fault Tolerance (PBFT) Blockchain Networks: Performance Analysis and Optimization

**DOI:** 10.3390/s24237688

**Published:** 2024-11-30

**Authors:** Ziyi Zhou, Oluwakayode Onireti, Lei Zhang, Muhammad Ali Imran

**Affiliations:** James Watt School of Engineering, University of Glasgow, Glasgow G12 8QQ, UK; z.zhou.3@research.gla.ac.uk (Z.Z.); lei.zhang@glasgow.ac.uk (L.Z.); muhammad.imran@glasgow.ac.uk (M.A.I.)

**Keywords:** PBFT, IoT network, consensus network, blockchain, wireless blockchain network, slotted ALOHA

## Abstract

Practical Byzantine Fault Tolerance (PBFT) is one of the most popular consensus mechanisms for the consortium and private blockchain technology. It has been recognized as a candidate consensus mechanism for the Internet of Things networks as it offers lower resource requirements and high performance when compared with other consensus mechanisms such as proof of work. In this paper, by considering the blockchain nodes are wirelessly connected, we model the network nodes distribution and transaction arrival rate as Poisson point process and we develop a framework for evaluating the performance of the wireless PBFT network. The framework utilizes slotted ALOHA as its multiple access technique. We derive the end-to-end success probability of the wireless PBFT network which serves as the basis for obtaining other key performance indicators namely, the optimal transmission interval, the transaction throughput and delay, and the viable area. The viable area represents the minimum PBFT coverage area that guarantees the liveness, safety, and resilience of the PBFT protocol while satisfying a predefined end-to-end success probability. Results show that the transmission interval required to make the wireless PBFT network viable can be reduced if either the end-to-end success probability requirement or the number of faulty nodes is lowered.

## 1. Introduction

About 500 billion devices will be connected to the Internet by 2030 and this represents a tenfold increase based on the 50 billion forecast for 2020 [1]. The traditional approach for implementing the Internet of Things (IoT) systems is the centralized architecture. In such an architecture, the IoT devices send information to the cloud server or a central entity, which then processes the information. The deployment of billions of IoT devices brings some fundamental design challenges with the centralized architecture in terms of privacy, security, confidentiality, scalability, slow operation, and high cost when third-party authentications are required [2,3].

As a fundamental technology for cryptocurrencies, blockchain technology can be used to address the security and trust vulnerabilities, and the high maintenance cost associated with the conventional IoT networks [4,5,6]. In particular, blockchain technology can be used in IoT device tracking, coordination and for processing transactions (The terminology “transactions” refers to any value information exchange between the nodes in the network) between devices. The decentralized blockchain network mitigates the risk of a single point of failure in centralized systems with nodes that transfer data in a peer-to-peer manner [6,7,8]. Moreover, an autonomous IoT network can be actualized when IoT devices or ‘nodes’ communicate in a distributed manner [9]. This requires a consensus method that allows nodes to agree on the validity of the message or communicated data. On the other hand, the blockchain system utilizes a consensus mechanism to agree on new data [10,11,12]. Hence, consensus mechanisms that are used in blockchain can also be applied to IoT networks. However, most IoT devices are constrained in terms of energy since they are battery-powered. They are also in limited bandwidth resources and computational capabilities. The aforementioned factors must be taken into consideration when selecting consensus mechanisms for IoT networks [8,11,13].

### 1.1. Practical Byzantine Fault Tolerance: A Consensus Mechanism for IoT Networks

The growth of Internet of Things (IoT) networks presents substantial challenges in maintaining data integrity, security, and fault tolerance across distributed, often resource-constrained, devices. Traditional centralized architectures struggle with scalability and single points of failure, making decentralized approaches, such as blockchain, appealing for IoT applications. Practical Byzantine Fault Tolerance (PBFT)—a blockchain consensus mechanism—offers low computational power and complexity, and it is well suited for IoT networks [13]. PBFT is a practical and improved protocol on Byzantine Fault Tolerance (BFT) that was proposed and implemented in [14], and it has better response time and peak throughput than BFT. It achieves these gains by incorporating several important optimizations, including reducing the size and number of messages, using message authentication codes, and integrating incremental checkpoint-management techniques. PBFT provides a significant reduction in energy consumption when compared with other blockchain consensus mechanisms such as PoW (proof of work) [15]. It is not affected by the “nothing at stake” and the centralization problems associated with PoS (proof of stake) [13]. The PBFT protocol guarantees safety and liveness when no more than $\left\lfloor \frac{n-1}{3}\right\rfloor$ out of the total *n* nodes are faulty. This implies that neither software or operator errors nor alterations by adversaries can cause a crash in the system when the number of faulty nodes is lower than this threshold.

PBFT has three main parts namely, the normal PBFT, the garbage collection, and the view change. The purpose of the normal operation is to ensure that the client’s (IoT device) requests are executed in a predefined order. It is the main procedure for making agreements among the nodes. The purpose of garbage collection is to recover in case of an accident. The view change purpose is to select a new primary (PBFT consensus mechanism selects one of the nodes in the network as the primary node while other nodes serve as backups) node and it takes effect when the primary in the consensus network either becomes a faulty node or breaks down. In the normal operation of the PBFT, when the primary node of the PBFT network receives the client request, it starts the three phases of the PBFT consensus mechanism namely, pre-prepare, prepare and commit. In the pre-prepare, the primary broadcasts a pre-prepare message to other nodes within the PBFT network. Moreover, in the prepare and commit phases, nodes communicate with each other using the broadcast protocol. By using these inter-node communications, the PBFT consensus mechanism ensures that all nodes are synchronized and agree that the transactions are legitimate, which are then added to the blockchain. PBFT consensus is thus critically important to blockchain systems since the consensus mechanism largely determines the blockchain system performance in terms of transaction throughput, delay, scalability, security, etc.

### 1.2. Motivation and Related Work

The majority of IoT devices rely on wireless communication protocols like WiFi and cellular networks, with blockchain integrated to enhance security and trust. Nevertheless, wireless communications between nodes face unique challenges due to inconsistent channel quality, network congestion, and inherent openness, which can impact the security, reliability, and performance of blockchain systems. Traditional PBFT (Practical Byzantine Fault Tolerance) networks generally assume ideal communication conditions, free from latency and bandwidth limitations. However, in wireless environments, fluctuating channel strength, restricted channel resources, and varying network topologies introduce complexities that must be addressed to understand how wireless communications affect PBFT-based blockchain network performance.

Previous research has explored alternative consensus mechanisms in wireless blockchain environments. For instance, Cao et al. [12] studied a Tangle consensus model in a wireless blockchain network. Further, Xu et al. [16] analyzed the security performance of Raft-based wireless blockchain systems. The authors in [4] developed a framework for evaluating blockchain transaction throughput and node placement in Proof-of-Work (PoW) based wireless networks. However, the analysis presented in [4,12,16] does not apply to wireless PBFT consensus mechanism due to its 3-phased operation and its peculiar inter-node communications.

Recently other BFT protocols such as scaled BFT (SBFT) [17], FastBFT [18] and HotStuff [19] have emerged. These new protocols have reduced communication cost while maintaining the safety, liveness and resilience properties of PBFT, thus making them more suitable for IoT networks. Though the analysis presented in this paper focuses on the wireless PBFT protocol, it can be easily extended to other wireless BFT protocols such as SBFT, FastBFT and HotStuff.

### 1.3. Contributions and Organization

In this paper, we propose a novel framework for implementing the PBFT consensus mechanism over a wireless channel. In particular, we consider that communications between the IoT nodes in the three phases of the normal operation of PBFT (i.e., pre-prepare, prepare and commit phases) are done over the wireless channel where node contend for channel access based on the slotted ALOHA protocol [20]. We derive the success probabilities over the prepare and commit phases of the wireless PBFT network for the case with non-faulty primary  (The pre-prepare phase success probability is equivalent to one since the primary node is non-faulty and there are no collisions in the pre-prepare phase). Besides, we derive the end-to-end success probability while considering an ideal pre-prepare and finishing phases  (The nodes send their reply to the client in the finishing phase). The end-to-end success probability serves as the basis for deriving other wireless PBFT key performance indicators (KPIs) namely, the transaction throughput which is expressed in terms of the number of transactions per second, transaction confirmation delay, the optimal transmission interval, and the viable area. We show the impact of view change as a result of the faulty primary by deriving the effective transaction confirmation delay. The transaction throughput demonstrates the transaction processing capacity of blockchain system and it’s maximized at the optimal transmission interval. This optimal interval also achieves the minimum end-to-end delay and it can be used to set the PBFT network timeouts. On the other hand, the viable area defines the minimum PBFT network’s coverage area required for a successful implementation of the PBFT consensus mechanism. In other words, the viable area ensures that the minimum number of nodes is activated in the wireless PBFT network, while satisfying all constraints. This will lead to significant savings in energy and an improvement in the overall performance. The viable area is decided by parameters such as the nodes’ transmit power and receiver sensitivity, transmission interval *v*, propagation channel parameters, success probability requirement and the number of faulty nodes *f*.

The main contributions of this work are summarized as follows.

- Considering the Spatio-temporal domain Poisson point process (PPP) modeling, i.e., node geographical distribution and transaction arrival rate in the time domain, we establish an analytical framework for obtaining the end-to-end success probability of the wireless PBFT network. Our framework provides a mathematical link between the wireless PBFT network’s coverage, the transmission interval, the nodes transmit power and the number of faulty nodes in the network. The proposed framework enables the optimization of network parameters such as the transmission interval, the end-to-end transaction throughput and delay, the broadcast transmit power and the coverage range.
- Using the framework, we define the viable area of the wireless PBFT network, which ensures that the minimum number of nodes are activated for a given end-to-end success probability requirement while guaranteeing the networks liveness, safety, and resilience. Furthermore, we present a low complexity algorithm for obtaining a viable area of the wireless PBFT network.
- We derive the effective transaction confirmation delay to show the effect of view change on the wireless PBFT network. Furthermore, numerical results are presented to verify our analytical derivations.

The rest of the paper is organized as follows. In Section 2, we present the system model and the description of the wireless PBFT algorithm. Section 3 presents the wireless PBFT network including its implementation constraints and the analysis of the independent prepare and commit success probabilities, and the end-to-end success probability. Section 4 presents the performance metrics of the wireless PBFT network namely, the average transaction throughput and delay, optimal transmission interval, effective transaction confirmation delay, average transmit power and the viable area. Numerical and simulation results are presented in Section 5. Finally, Section 6 concludes the paper. A preliminary version of this paper has been reported in [21] where we introduced the viable area concept for the wireless PBFT network without considering the effect of channel contention. Herein, our analysis has been made more generic and realistic with the inclusion of the effect of channel contention. Furthermore, new performance metrics such as the end-to-end success probability and the average transaction throughput have been derived. A detailed analysis of the optimal transmission interval has also been conducted.

## 2. System Model and Assumptions

In this section, we present the wireless PBFT system model and algorithmic description.

### 2.1. Practical Byzantine Fault Tolerance (PBFT)

We consider the weak-synchronous, distributed system where the IoT devices (nodes) are connected via a wireless network. The target of the PBFT consensus mechanism is to achieve agreement on a transaction as only agreed transactions can be added to the blockchain. We utilize the Byzantine failure model where the faulty nodes may behave arbitrarily. Further, node failures in the network are independent of each other. A wireless PBFT consensus network that is made up of *n* nodes can tolerate a maximum of *f* faulty/Byzantine  (A Byzantine node is a tyrant node that can intentionally mislead other nodes in the consensus mechanism) nodes where the relationship between *f* and *n* as defined in [14] is such that

(1) $$f\leq \left\lfloor \frac{n-1}{3}\right\rfloor .\;$$

In other words, agreement on a transaction can only be reached when the expression in (1) is satisfied. A consensus mechanism should be able to overcome the intervention by Byzantine nodes. Moreover, wireless PBFT must guarantee the following two attributes of distributed systems [22]:

- Safety: all non-faulty nodes execute the requests in the same order (i.e., consensus);
- Liveness: client eventually receive replies to their requests.

To ensure liveness, PBFT uses weak synchrony assumption that messages are guaranteed to be delivered after a certain time bound to circumvent FLP impossibility result [23]. The wireless PBFT consensus algorithm provides liveness and safety when not more than $\left\lfloor \frac{n-1}{3}\right\rfloor$ nodes are faulty. Moreover, the resilience of the PBFT consensus network is a measure of its ability to provide both safety and liveness. The optimal resiliency of PBFT is achieved at n=3f+1. The two main reasons for this are: (1) the consensus network can proceed after communicating with n−f (i.e., 2f+1) nodes considering that up to *f* nodes might not respond due to channel quality, congestions, etc, and (2) Consensus can still be achieved even if up to *f* of the responding n−f nodes are faulty. Note that the other f+1 which are not faulty achieves the majority in a consensus vote. The difference between the conventional and the wireless PBFT consensus network is the wireless nature of the channel which encompasses the channel propagation effect, channel congestion, channel collision, interference, etc.

### 2.2. PBFT Algorithm

**Figure 1:**
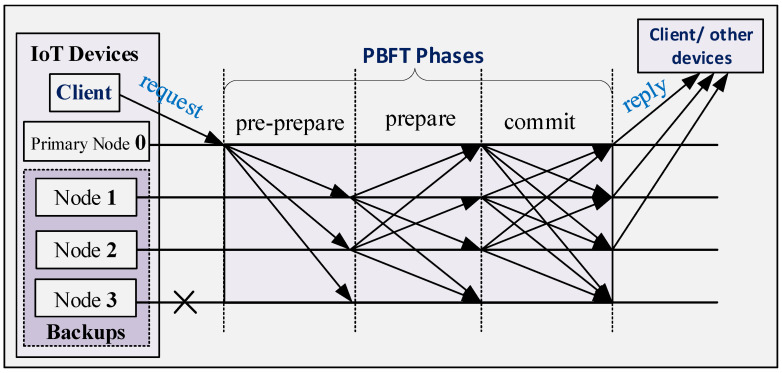
Normal case operation of the Practical Byzantine Fault Tolerance (PBFT) network [14].

We represent the set of nodes by G and identify each node by utilizing an integer in {0,1,…,|G|−1}. Note that we could have more than 3f+1 nodes for the PBFT consensus mechanism, however, the additional nodes degrade the network performance since the additional messaging does not lead to an improvement in resilience. The network moves through a succession of configurations called views as it progresses, and for each view, it selects one of the nodes as the primary node while other nodes serve as backups, as illustrated in Figure 1. The nodes can take turns to be the primary node. The primary for the view *w* is denoted by $w_{p}$ which can be obtained as follows:

(2) $$w_{p}=w\;\left(\mathrm{mod}\;n\right)$$

The operational steps of the wireless PBFT consensus network, which is illustrated in Figure 1, are as follows [14]:

- Client sends a service request to the primary of the PBFT network through a wireless link using the unicast transmission protocol.
- The primary node validates the request and initiates PBFT’s three phases protocols namely, the pre-prepare, prepare and commit  (Here we focus on the wireless communication aspect. Details of the authentication process, content of the messages, clock, and order in each of the phases can be found in [14,22].). Each of the phases is implemented over the wireless link while using the broadcast transmission protocol.
- Each node executes the requests and send the result directly to the client via a wireless link using the unicast transmission protocol.
- The client node waits for f+1 replies from different nodes with the same response message.

Our framework considers a client as an IoT device that exchange information or makes a transaction with other IoT devices referred to as nodes. The client’s transactions are recorded on the blockchain once consensus is reached. The framework considers that the client node waits for the completion of one request before sending the next request.

## 3. Wireless PBFT Network

**Figure 2:**
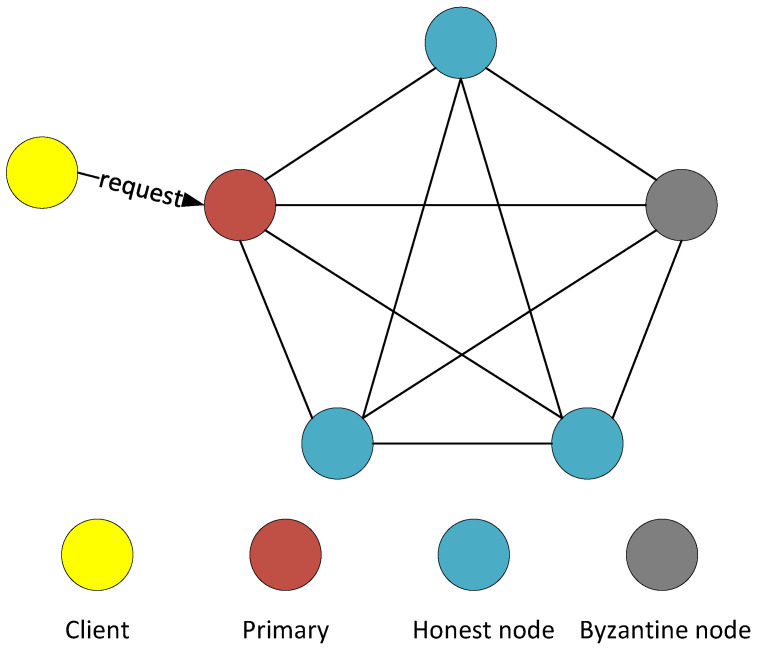
Spatial distribution of the Practical Byzantine Fault Tolerance (PBFT) network.

IoT is one of the main application scenarios of blockchain [24], and IoT nodes could be connected wirelessly. Considering the unstable channel quality, interference, limited resource, and various network topology [25], it is necessary to investigate the impact of wireless communications on the PBFT-based blockchain networks. We consider that the nodes are spatially distributed in $R^{2}$ according to a homogeneous PPP with density λ. Furthermore, we consider a noise-limited wireless network where all nodes have the same receiver sensitivity $\beta_{1}$. In the first phase of the wireless PBFT consensus system, the primary node broadcasts a pre-prepare message to the whole network over the wireless channel, as illustrated in Figure 2. We consider that all nodes are equipped with an omnidirectional antenna. Hence, the coverage range of the primary node can be expressed from [26] as

(3) $$R_{1}=d_{0}{\left[\frac{P_{1}V}{\beta_{1}}\right]}^{\frac{1}{\gamma }},$$

where $P_{1}$ is the transmit power of the primary node. Note that the coverage range is based on the maximum long-term averaged channel power, i.e., the effect of the channel fading has been averaged out. The parameter γ is the pathloss exponent, $d_{0}$ is a reference distance for the antenna far-field, and *V* is a unit-less constant that depends on the antenna characteristics and the average channel attenuation. The broadcasted pre-prepare message by the primary node will be received by all other nodes within its coverage radius, i.e., $R_{1}$. These nodes then perform the necessary verification to ascertain the validity of the block, as shown in Figure 1. The number of nodes within the coverage of the primary can be obtained from the following approximation

(4) $$n=\lfloor \lambda K\left(R_{1}\right)\rfloor ,$$

where $\lambda \;K(R_{1})$ is the expected number of other nodes within a circle of radius $R_{1}$ centered on a typical point of the process, i.e. the primary node. Further, the *K* function $K\;\left(R_{1}\right)\;=\;\pi \;R_{1}^{2}$ for $R^{2}$.

**Constraint 1**

In the wireless PBFT consensus network with *f* faulty nodes, the number of nodes within the primary node’s coverage, which is denoted by *n*, must satisfy the expression in (1). The primary node’s coverage, which is also within the network’s coverage, can be defined as $A_{1}\equiv K\left(R_{1}\right)$. Hence, the constraint can be expressed as follows:

$$n=\lfloor A_{1}\lambda \rfloor \geq 3f+1.$$

Each node then enters the prepare stage after accepting the pre-prepare, as illustrated in Figure 1. Note that a larger primary node coverage will increase the safety of the network but this also leads to an increase in the energy consumption and besides, an increase in the likelihood of collision of messages broadcasted by the nodes in the prepare and commit phases.

### 3.1. Channel Contention in Wireless PBFT Consensus Network

**Figure 3:**
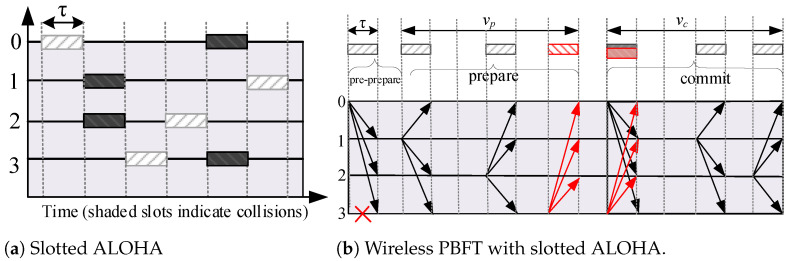
Illustration of channel contention in wireless PBFT.

We utilize the slotted ALOHA as the medium access protocol for the wireless PBFT-based blockchain network. Moreover, LoRa is a low-power wide-area network (LPWAN) technology that has been specifically developed for IoT and it uses slotted ALOHA as its multiple access technique [27]. In this model, we consider the PBFT nodes to be class B, and the primary may act as the central server. The length of the receiving window for the nodes is supposed to be slightly more than the combination of the timers of the prepare and commit phases. When the primary node receives a request from the client, it broadcasts a beacon to the other nodes to trigger the receive windows.In slotted ALOHA, time is divided into slots of size τ and nodes start to transmit frames/messages only at the beginnings of slots. A collision occurs if two or more nodes transmit their message or packet at the same slot. Otherwise, there are no collisions and packets are properly sent as illustrated in Figure 3a. Message broadcast in wireless PBFT network is done over the wireless channel where nodes contend with each other for channel access. We assume that packets are lost when there are collisions over the channel such that the sent messages are not received by other nodes in the network. Consequently, collisions have a negative impact on the liveness, safety, and resilience of the wireless PBFT consensus network. In particular, all nodes need to contend the same channel, thus collisions can occur which could then lead to a less secured consensus network.

The prepare and commit success probabilities and end-to-end success probability of the conventional PBFT network relies on the number of Byzantine/faulty nodes and the total number of nodes in the network. In addition to these two elements, the wireless PBFT consensus network must incorporate the effect of packet collision as well when evaluating its success probabilities.

### 3.2. Wireless PBFT Success Probability

In the following, we analyze the success probabilities of each phase of the wireless PBFT network and its end-to-end success probability.

#### 3.2.1. Pre-Prepare Phase

There are no channel contention or collisions in the pre-prepare phase since only the primary node accesses the channel. Hence, the broadcasted pre-prepare message by the primary is successfully received and decoded by all nodes that are within the primary’s coverage as defined by (3). Here we assume that the primary node of the current view is non-faulty. A faulty primary will warrant a change of view, i.e., selection of a new primary node.

#### 3.2.2. Prepare Phase

Given that n−1 nodes accept the pre-prepare message from the primary, then each node enters the prepare phase by broadcasting the prepare message to all other nodes. Hence, n−1 nodes contend for channel access during the prepare phase. Note that the primary (denoted by 0) does not broadcast any message during the prepare phase, as illustrated in Figure 1 and Figure 3b. Here we assume that each prepare message is contained in a packet of length τ, which is equivalent to the slot size, and that the population of nodes attempts to broadcast according to a Poisson distribution. Let the interval $v_{p}$ denote the time frame between the first packet and the last packet in the prepare phase, as illustrated in Figure 3b. Consequently, the traffic generated in the interval $v_{p}$ can be expressed as

(5) $$G_{p}=\frac{(n-1)\tau }{v_{p}}.$$

Considering the contention of nodes without the PBFT constraints in the prepare phase, the throughput in the prepare phase can be obtained as the conventional slotted ALOHA throughput $G_{p}e^{-G_{p}}$, i.e., the product of the rate of transmission $G_{p}$ and the probability of success $e^{-G_{p}}$ [20]. In that case, the optimal traffic load, $G_{p}^{★}=1$, such that the optimal interval $v_{p}^{★}$ can be expressed as $v_{p}^{★}=\left(n-1\right)\tau$. For non-faulty nodes to attain the prepared state in the wireless PBFT network, at least 2f transmissions by the nodes in the prepare state must be successful [14]. Upon defining the probability of generating *i* successful transmission over n−1 contending node in a generic slot:

(6) n−1ie−Gpi1−e−Gpn−1−i

the success probability in the prepare phase can be obtained from the following Lemma.

**Lemma** **1.** *The success probability of the prepare phase of wireless PBFT network with slotted ALOHA access protocol can be expressed as*

(7) Pp=0n<2f∑i=2fn−1n−1ie−Gpi1−e−Gpn−1−in≥2f,

*where $G_{p}$ is defined in (5).*

**Proof.**  To become prepared, a non-faulty node should have 2f prepare messages from different nodes that match the pre-prepare message from the primary node. Here we consider that the primary of the current view is non-faulty and that only the primary node transmits in the pre-prepare phase. Consequently, the transmission by the primary node in the pre-prepare phase will be successfully received by all nodes within its coverage. Now consider that each node broadcast its prepare message once and that there is no acknowledgement (ACK), request to send (RTS) or clear to send (CTS) messages due to the broadcast nature. Consequently, the number of transmissions in the prepare state is equivalent to n−1, i.e., the number of nodes excluding the primary. Note that only prepare message received from faulty nodes will not match with the pre-prepare from the primary. Further, since a log of 2f prepare messages from different nodes are required, the minimum number of nodes with successful transmission in the prepare phase is also set to 2f. Hence, the starting point of the summation in (7) is 2f, and $P_{p}=0;\forall n<2f$. Note that *f* out of the 2f (required minimum number of successful transmissions in prepare phase) successful transmissions could be transmissions from Byzantine or faulty nodes. Nevertheless, the prepared state will still be attained by each of the non-faulty nodes in this case since the received transmissions from the other *f* nodes will match with the pre-prepare and achieve a majority of f+1 as against the *f* Byzantine broadcasts in the prepare consensus. The resilience of the PBFT consensus protocol is thus preserved by restraining the minimum number of successful transmissions in the prepare phase of the wireless PBFT to 2f, irrespective of whether Byzantine nodes’ transmissions have suffered from collision or not.    □

**Assumption (Non-Overlapping Prepare and Commit Phases)**

We assume that all non-faulty nodes are prepared at approximately the same time instance such that traffic generated during the prepare phase does not overlap with the commit phase.

In Lemma 1, all nodes that are within the primary’s coverage broadcast a prepare message within the time-frame denoted $v_{p}$, and successful transmission from each node are received and decoded by all other nodes within the primary’s coverage. Consequently, all non-faulty nodes will be prepared, i.e., receive 2f prepare messages that match with the pre-prepare, at approximately the same time instance including non-faulty nodes that are yet to broadcast their own prepare message. Prepared non-faulty nodes thus move directly to the commit phase at the same time instance, and hence, *the prepare and commit phases of wireless PBFT consensus mechanism are non-overlapping*. For tractability sake and to retain the resilience associated with the conventional PBFT protocol, the commit phase commences when $v_{p}$ elapses, as shown in Figure 3b.

#### 3.2.3. Commit Phase

The operation of the commit phase in the wireless PBFT network is very similar to the prepare phase. Considering that $\bar{n}$ nodes were successful in the prepare stage where $\bar{n}\leq n-1$, then $\bar{n}+1$ nodes, i.e., all successful nodes in the prepare phase and the primary node will contend for channel access in the commit phase. Each node broadcasts a commit message to other nodes when its prepared becomes true. We denote the interval between the first packet and the last packet in the commit phase by $v_{c}$ such that the traffic generated can be expressed as

(8) $$G_{c}=\frac{\left(\bar{n}+1\right)\tau }{v_{c}}.$$

Similar to the prepare phase, the success probability in the commit phase can be obtained from the following Lemma.

**Lemma** **2.** *The success probability of the commit phase of wireless PBFT network with slotted ALOHA access protocol can be expressed as*

(9) Pc=0n¯<2f∑i=2f+1n¯+1n¯+1ie−Gci1−e−Gcn¯+1−in¯≥2f,

*where $G_{c}$ is defined in (8).*

**Proof.**  The minimum number of successful transmissions for a successful commit is 2f+1, which is the starting point of the summation in (9) such that the commit success probability $P_{c}=0,\forall \;\bar{n}<2f$. The rest of the proof follows directly from Lemma 1.    □

#### 3.2.4. End-to-End Success Probability

Given that the primary is a non-faulty node, the success probability of the pre-prepare phase is thus equivalent to one since there are no channel contention or collisions in the pre-prepare phase. Moreover, the success of the commit phase is dependent on the success of the prepare phase. Considering that the finishing state i.e., “reply to the client” by committed nodes is ideal (i.e., all successful commits are successfully reported to the client), the end-to-end success probability of the wireless PBFT network is given in the following Theorem.

**Theorem** **1.** *The end-to-end success probability of the wireless PBFT network with slotted ALOHA access protocol can be expressed as*

(10) Ps=0n<2f+1∑i=2fn−1∑x=2f+1nn−1inxe−Gpi(1−e−Gp)n−1−ie−Gcx(1−e−Gc)n−xn≥2f+1.

**Proof.**  The proof follows from the dependence of the commit phase on the prepare phase, and Lemmas 1 and 2.    □

From (10), it can be seen that for n>2f+1, the end-to-end success probability $P_{s}\to 1$ when $v_{c}\gg n\tau$ and $v_{p}\gg \left(n-1\right)\tau$.

**Corollary** **1.** *The end-to-end success probability of the wireless PBFT with the same transmission interval $v_{p}=v_{c}=v$ for the prepare and commit phases can be computed as*

(11) Ps=0n<2f+1∑i=2fn−1∑x=2f+1nn−1inxe−(n−1)i+nxτv(1−e−(n−1)τv)n−1−i(1−e−nτv)n−xn≥2f+1.

Note that the end-to-end success probability $P_{s}$ is the probability that the transaction initiated by the client IoT device is agreed on by the nodes in the wireless PBFT consensus mechanism and added to the blockchain. The end-to-end success probability is thus a very important metric in the wireless PBFT network as it directly relates to KPIs such, as the transaction throughput and the end-to-end delay. In the next section, we utilize the success probability of the wireless PBFT network as the basis for deriving other KPIs.

## 4. Performance Metric of Wireless PBFT Networks

In this section, we derive the KPIs for the wireless PBFT consensus networks namely, the average transaction throughput, optimal transmission interval, transaction confirmation delay, average transmit power and the viable area.

### 4.1. Average Prepare/Commit Transaction Throughput and Optimal Interval

The transaction throughput can be defined as the number of transactions confirmed in a unit time by the PBFT consensus mechanism. Here we use transactions per second as the metric. The transaction throughput can be used to demonstrate the transaction processing capacity of the consensus mechanism. Given the transmission interval $\bar{v}$ for the independent prepare and commit phases, the average independent prepare/commit transaction throughput of the wireless PBFT network can be expressed as

(12) T(v¯)=1v¯P=1v¯∑i=INNie−Nτiv¯1−e−Nτv¯N−i,

where $\bar{v}=v_{p},P=P_{p},I=2f$ and N=n−1 for the prepare phase, while $\bar{v}=v_{c}$, $P=P_{c},I=2f+1$ and $N=\bar{n}+1$ for the commit phase. The independent prepare and commit success probabilities, i.e., $P_{p}$ and $P_{c}$ are defined in (7) and (9), respectively. Consequently, the optimal transmission interval for each phase can be obtained by finding ${\bar{v}}^{★}$ that maximizes the average independent transaction throughput $T(\bar{v})$ in (12). $T(\bar{v})$ as defined from (12) is differentiable over its domain, i.e. for $\bar{v}\in \left[I\tau ,{\bar{v}}_{\max}\right]$ such that $\frac{\partial T(\bar{v})}{\partial \bar{v}}$ can be expressed after simplification as

(13) ∂T(v¯)∂v¯=∑i=INNie−Nτiv¯1−e−Nτv¯N−i×Nτiv¯3−N−iNτe−Nτv¯v¯31−e−Nτv¯−1v¯2.

Note that Iτ is the minimum possible transmission interval for a successful prepare and commit phase while ${\bar{v}}_{\max}$ is a system-defined parameter. Let ${\bar{v}}^{★}$ be the solution to the equation $\frac{\partial T(\bar{v})}{\partial \bar{v}}=0$. Then $\frac{\partial T(\bar{v})}{\partial \bar{v}}\geq 0$ and $\frac{\partial T(\bar{v})}{\partial \bar{v}}\leq 0$ for any $\bar{v}\in \left[I\tau ,{\bar{v}}^{★}\right]$ and $\left[{\bar{v}}^{★},v_{\max}\right]$, respectively, which in turn implies that $T(\bar{v})$ increases over $\bar{v}\in \left[I\tau ,{\bar{v}}^{★}\right]$ and decreases over $\left[{\bar{v}}^{★},v_{\max}\right]$. Consequently, $T(\bar{v})$ has a unique maximum, which occurs at $\bar{v}={\bar{v}}^{★}$. By setting $\frac{\partial T(\bar{v}={\bar{v}}^{★})}{\partial \bar{v}}=0$, the optimal interval ${\bar{v}}^{★}$ can be obtained from (13) with the use of linear search methods.

### 4.2. Average End-to-End Transaction Throughput and Optimal Interval

We consider the same interval *v* for the prepare and commit phases, and that the transmission intervals are reserved irrespective of the success of the phases. Further, the transmission interval for the pre-prepare phase $v_{pp}=\zeta \tau$ since there is no contention in the pre-prepare phase and the pre-prepare broadcast can be synced with the transmission slot of the slotted ALOHA protocol in the pre-prepare phase. The parameter ζ denotes the tolerance interval [28]. Consequently, the average end-to-end transaction throughput for the wireless PBFT network, i.e., the average number of successful transactions per unit second, can be expressed as

(14) Ts(v)=12v+ζτPs=12v+ζτ∑i=2fn−1∑x=2f+1nn−1inxe−(n−1)i+nxτv×1−e−(n−1)τvn−1−i1−e−nτvn−x,

where $P_{s}$ is the end-to-end success probability which has been defined earlier in (11). Following the same reasoning as in the case of an independent prepare or commit phase, there exists an optimal transmission interval $v^{★}\in \left[I\tau ,v_{\max}\right]$ that maximizes the end-to-end transaction throughput $T_{s}\left(v\right)$. The optimal transmission interval $v^{★}$ can be obtained by solving the expression $\frac{\partial T_{s}\left(v\right)}{\partial v}=0$ which upon simplification can be expressed as

(15) $$\frac{\partial T_{s}\left(v\right)}{\partial v}=\sum_{i=2f}^{n-1}\sum_{x=2f+1}^{n}a_{1}^{\prime }a_{2}a_{3}a_{4}+a_{1}a_{2}^{\prime }a_{3}a_{4}+a_{1}a_{2}a_{3}^{\prime }a_{4}+a_{1}a_{2}a_{3}a_{4}^{\prime },$$

where $a_{1}=\frac{1}{2v+\zeta \tau },a_{2}=e^{-\frac{\left((n-1)i+nx\right)\tau }{v}},a_{3}={\left(1-e^{-\frac{(n-1)\tau }{v}}\right)}^{n-1-i},a_{4}={\left(1-e^{-\frac{n\tau }{v}}\right)}^{n-x}$, and $a_{n}^{\prime }\forall n\in \left\{1,\ldots ,4\right\}$ denotes the differential of $a_{n}$. Similarly, the optimal transmission interval which also corresponds to the maximum end-to-end transaction throughput can be obtained from (15) with the use of linear search algorithms.

### 4.3. Transaction Confirmation Delay

The transaction confirmation delay $D_{s}$ from client IoT device to the stage of being confirmed by the wireless PBFT consensus mechanism can be expressed from [12] as

(16) $$D_{s}=D_{q}+D_{a},$$

where $D_{q}$ is the delay from the time that the transaction arrives at the client’s cache to the time it is received by the primary node of the wireless PBFT network, as illustrated in Figure 1. $D_{a}$ is the delay as a result of the 3-phased (pre-prepare, prepare and commit phases) wireless PBFT consesus mechanism and it can be calculated as the inverse of the end-to-end throughput, i.e., $D_{a}=\frac{1}{T_{s}}\left(v\right)$. The transaction confirmation delay thus estimates the average time for a transaction to be confirmed by the PBFT consensus network.

### 4.4. Impact of PBFT View Change on the Transaction Confirmation Delay

The view-change protocol provides liveness to the wireless PBFT network by allowing the system to make progress when the primary node fails [14]. View changes uses timeouts to prevent nodes from waiting indefinitely for requests to execute in PBFT. In the following, we analyse the effect of the view change operation on the performance of the wireless PBFT network. After a timeout defined by $\zeta_{1}$ without replies, the client broadcasts the message to all nodes. In this case, a node that has already committed resends the message while nodes that are still processing ignores the client message. Nodes that have not received the pre-prepare forwards the message to the primary node. If a node does not receive the pre-prepare message back after a certain time $\zeta_{2}$, it decides that the primary node is Byzantine/faulty. A view change is thus initiated [14]. After the *i*th node has received 2f view change message from the other nodes in the PBFT network, it broadcast a new view message that contains the signed view change. Each node then verifies the signature and this is followed by accepting the view change to the new primary node.

Meanwhile, in a wireless PBFT network, the new primary node could also be faulty as well. If this happen another timeout is used in the view-change. At the expiration of the timeout, another node will be chosen as the primary. Noting that there are at most *f* faulty nodes, the primary node can be faulty for at most *f* consecutive times. As a result of the view change the effective transaction confirmation delay $D_{e}$ will exceed $D_{s}$ obtained earlier in (16). The following Theorem gives the effective transaction confirmation delay of wireless PBFT with view-change.

**Theorem** **2.** *The effective transaction confirmation delay of the wireless PBFT network with transaction confirmation delay $D_{s}$ can be expressed as*

(17) $$D_{e}\;=\;\left\{\;\;\begin{array}{cc} \frac{N-1}{N}D_{s}+\frac{1}{N}\left(\zeta +D_{s}\right) & f=1\\ \frac{N-f}{N}D_{s}\;+\;\frac{f}{N}\;\left(\;\zeta \;+\;\left(\frac{N-F}{N-1}\right)D_{s}\;+\;\sum_{i=2}^{F}\left[\left(i-1\right)\zeta +D_{s}\right]\frac{N-F}{N-i}\prod_{v=1}^{i-1}\left(\frac{F-v}{N-v}\right)\right) & f>1 \end{array}\right.,$$

*where $\zeta =\zeta_{1}+\zeta_{2}$ is the total timeout for each view change.*

**Proof.**  In a wireless PBFT network with *f* faulty nodes, the primary node is selected according to (2). There exist a probability $\frac{f}{N}$ that the primary of a view is faulty. Each view-change occurs after a timeout ζ. For the *i*th consecutive selection of a faulty primary there also exist a probability $\frac{N-F}{N-i}$ that the next selected node is non-faulty. Further, after *f* consecutive selection of a faulty node, the probability of selecting a non-faulty node is 1. The effective transaction confirmation delay can thus be obtained by averaging the transaction confirmation delay and timeouts over the probabilities.    □

### 4.5. Average Transmit Power of a Typical Node in the Wireless PBFT Network

To guarantee the resilience of the wireless PBFT consensus network, each node must be able to transmit and receive from n=3f+1 nodes since *f* nodes can suffer from a collision and their transmission might not be received by the other nodes. Thus leaving 2f+1 nodes with a successful transmission out of which *f* (successful transmission) could be from Byzantine nodes. In other words, the wireless PBFT network with n=3f+1, non-faulty nodes will achieve the prepared and committed states even when transmitted packet by *f* nodes suffer from collision (i.e. transmitted prepare or commit message fails to reach other nodes) and *f* other nodes are Byzantine. Each node adjusts its transmit power such that its transmission will be successfully received by other nodes in the network. Given that the primary node located at the origin has a coverage range $R_{1}$ defined in (3), the *i*th node at a distance $r_{i}$ from the primary must set its transmit power to

(18) $$P_{2,i}\geq \frac{\beta_{1}}{V}{\left(\frac{R_{1}+r_{i}}{d_{0}}\right)}^{\gamma }$$

to guarantee the resilience associated with the conventional PBFT protocol. The probability distribution function for the distance $r_{i}$ between the *i*th node located in the circular area with radius $R_{1}$ centered at the primary’s location, $f_{{R_{1}}^{\left(i\right)}}\left(r^{\left(i\right)}\right)$, can be expressed as

(19) $$f_{{R_{1}}^{\left(i\right)}}\left(r^{\left(i\right)}\right)=\frac{2r^{\left(i\right)}}{{\left(R_{1}\right)}^{2}}.$$

Consequently, the average transmit power of a typical node that is within the coverage of the primary can be obtained as

(20) $${\bar{P}}_{2}\geq \int_{0}^{R_{1}}\frac{\beta_{1}}{V}{\left(\frac{R_{1}+r}{d_{0}}\right)}^{\gamma }f_{R_{1}}\left(r\right)dr,$$

which simplifies as

(21) $${\bar{P}}_{2}\geq \frac{2\beta_{1}R_{1}^{\gamma +2}\left(\gamma 2^{\gamma +1}+1\right)}{R_{1}^{2}Vd_{0}^{\gamma }\left(\gamma^{2}+3\gamma +2\right)}=\frac{2\beta_{1}R_{1}^{\gamma }\left(\gamma 2^{\gamma +1}+1\right)}{Vd_{0}^{\gamma }\left(\gamma^{2}+3\gamma +2\right)},$$

where γ≥2. Note that the average transmit power of a typical node ${\bar{P}}_{2}$ is greater than the primary’s transmit power, i.e., ${\bar{P}}_{2}>P_{1}$ since $0<r_{i}\leq R$ and nodes are uniformly distributed within the primary’s coverage.

### 4.6. Viable Area of Wireless PBFT Network

The wireless PBFT network coverage area is limited by the primary’s coverage and in particular, the primary’s transmit power since all nodes within the primary’s coverage receive the pre-prepare message which initiates the prepare and then the commit phase. The end-to-end success probability of the wireless PBFT network $P_{s}$ defined (11) is a function of the number of nodes in the network *n*, the number of faulty or Byzantine nodes *f* and the transmission interval *v*. The end-to-end success probability $P_{s}\to 1$ as the transmission intervals for the prepare and commit phases v→∞ when n≥3f+1. However, since it is not realistic to have an infinite transmission interval, we define the viable area for a wireless PBFT consensus network given an end-to-end success probability requirement that must be met by the network. The viable area ensures that the minimum number of nodes is activated for a given end-to-end success probability requirement $P_{s}$, number of faulty nodes *f*, and transmission interval *v*. This will lead to significant savings in energy and an improvement in the overall performance.

**Definition**

The viable area of the wireless PBFT network with node density λ, *f* Byzantine nodes, and transmission interval *v* is the minimum PBFT coverage area (or equivalently the minimum number of nodes) that meets *constraint 1* in Section 3 while satisfying a predefined end-to-end success probability $P_{s,th}$. The viable area can be obtained by solving the following optimization problem

(22) minimizenλA1(n)s.t.n≥3f+1∑i=2fn−1∑x=2f+1nn−1inxe−(n−1)i+nxτv×(1−e−(n−1)τv)n−1−i(1−e−nτv)n−x≥Ps,th

Considering that the primary node is located at the origin and the circular area $A_{1}$ is centered at the origin, the number of nodes within the viable area can be approximated as $n=\lfloor \lambda A_{1}\rfloor$. Further, the primary’s transmit power $P_{1}$ and equivalently the number of active nodes in the wireless PBFT network can be tuned to achieve a viable area. The first constraint in (22) is a requirement for the conventional PBFT network [14]. Note that for a fixed interval, having more active nodes can increase the collision probability and thus reduce the end-to-end success probability. On the other hand, increasing the density of non-faulty nodes can as well increase the end-to-end success probability of the PBFT consensus network. The second constraint thus ensures that the number of active nodes satisfies the end-to-end success probability requirement. Algorithm 1 describes a simple exhaustive search algorithm used to solve the optimization problem defined in (22). Note that the primary node transmit power $P_{1}$ and the optimal number of nodes in the viable area of the wireless PBFT network $n^{★}$ are such that $P_{1}^{★}={\left(\frac{n^{★}}{\pi \lambda \kappa }\right)}^{\frac{\gamma }{2}}$. The viable area of the wireless PBFT network thus defines the optimal settings for the PBFT network as it achieves the user or network pre-defined end-to-end success probability without sacrificing the safety, liveness, and resilience of the consensus protocol.
**Algorithm 1** Viable Area of Wireless PBFT Network    **Input**: $i=0,v,f,n_{0}^{i}=3f+1,P_{s}^{0}=P_{s}\left(v,n_{0}^{i}\right),P_{s,th}$   **Output**: $n^{★}$1:**if** $P_{s}^{0}\geq P_{s,th}$ **then**2:   $n^{★}=n_{0}^{i}$3:**else**4:   $i=i+1,n_{0}^{i}=n_{0}^{i}+1,P_{s}^{i}=P_{s}\left(v,n_{0}^{i}\right)$5:   **if** $P_{s}^{i}\geq P_{s,th}$ **then**6:     $n^{★}=n_{0}^{i}$7:   **else if** $P_{s}^{i}>P_{s}^{i-1}$ **then**8:     **go to** 49:   **else**10:     $n^{★}=0$11:   **end if**12:**end if**

## 5. Numerical Results

In this section, we present some numerical results to illustrate our analytical findings. The system parameters are as follows: $V=1,d_{0}=1,\beta_{1}=-84.5\;\mathrm{dBm}$, $\beta_{2}=-84.5\;\mathrm{dBm},\lambda =\frac{1}{\pi 1000}\;\mathrm{nodes}/m^{2}$ and γ=4. Regarding the broadcast message, the channel bit rate is 1Mbit/s, the payload length is 8184bits while the MAC and PHY header lengths are 272bits and 128bits, respectively, such that τ=8.584ms [29]. We set the PBFT coverage radius R=1000m and show results for the wireless PBFT consensus networks.

In this section, we evaluate the performance of the wireless PBFT network in terms of the optimal transmission interval $v^{★}$, the end-to-end success probability $P_{s}$ and the end-to-end transaction throughput $T_{s}$. We consider that the primary node is located at the origin and its coverage range $R_{1}$ (and equivalently the primary node’s transmit power $P_{1}$) is obtained by assuming that all nodes are with the same receiver sensitivity $\beta_{1}$. $P_{1}$ and equivalently $R_{1}$ is defined such that there are n=3f+1 nodes within the coverage of the primary. Note that $P_{1}$ required to achieve *n* can be adjusted based on the node density λ, however, the latter is fixed in our evaluations unless otherwise stated. From (3) and (5) the primary node’s transmit power $P_{1}$ and the number of nodes in the wireless PBFT network *n* is such that $P_{1}={\left(\frac{n}{\pi \lambda \kappa }\right)}^{\frac{\gamma }{2}}$.

**Figure 4:**
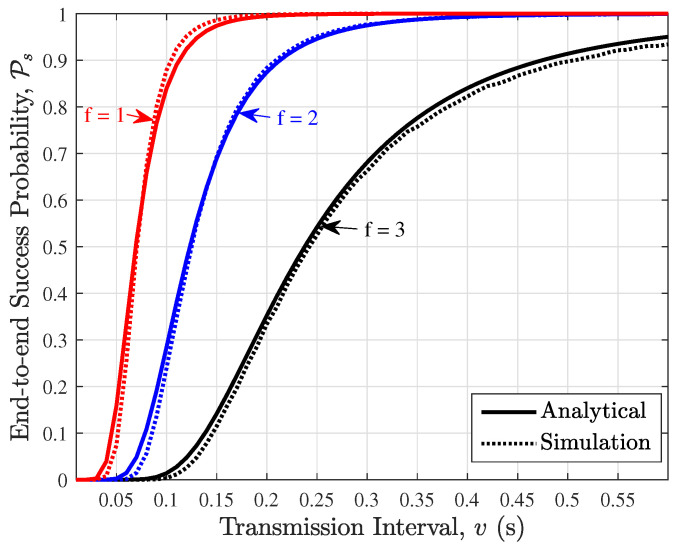
End-to-end success probability $P_{s}$ plotted against the transmission interval for the prepare and commit phases $v_{p}=v_{c}=v$).

In Figure 4, we compare the derived end-to-end success probability with simulation for the wireless PBFT network with n=10 nodes and f=1,2,3 faulty nodes. Results show a tight match between the analytical derivation and simulation results. As expected, it can be seen that for a fixed transmission interval, increasing the number of faulty nodes *f* leads to a reduction in the end-to-end success probability since the likelihood of reaching the prepared or committed state reduces as *f* increases. On the other hand, for a fixed number of faulty nodes *f*, increasing the transmission interval increases the end-to-end success probability. This is due to the fact that the probability that transmitted prepare/commit messages/packets encounter a collision reduces with increasing transmission interval *v*.

**Figure 5:**
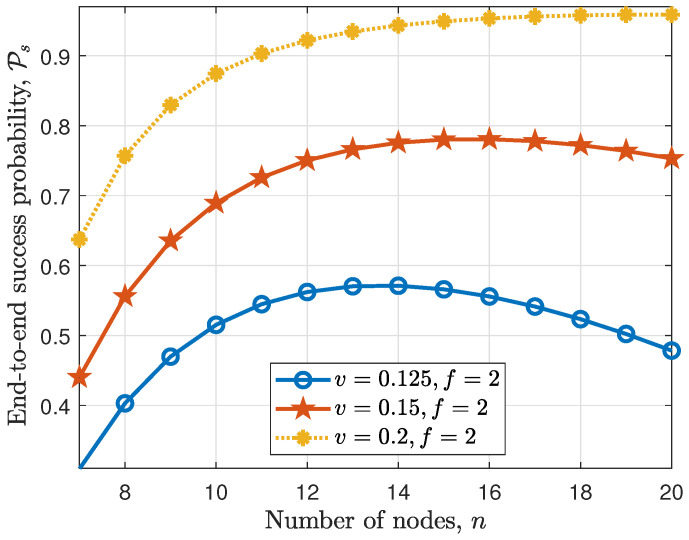
Effect of increasing the number of nodes *n* and the transmission interval *v* on end-to-end success probability.

In Figure 5, we show the effect of increasing the number of nodes in a fixed area (i.e., increasing the node density λ) for f=2 and transmission interval v=0.125,0.15,0.2s. It can be seen that when the transmission interval is high enough such as v=0.2s, increasing the number of nodes lead to an increase in the end-to-end success probability. This is due to the fact that the probability of a node receiving prepare or commit messages from non-faulty nodes increases as the node density increases. Moreover, when a particular number of nodes is reached, the effect of the collision of the nodes’ transmissions becomes more pronounced, and the end-to-end success probability starts to decrease as seen for the case with the transmission interval v=0.125s.

**Figure 6:**
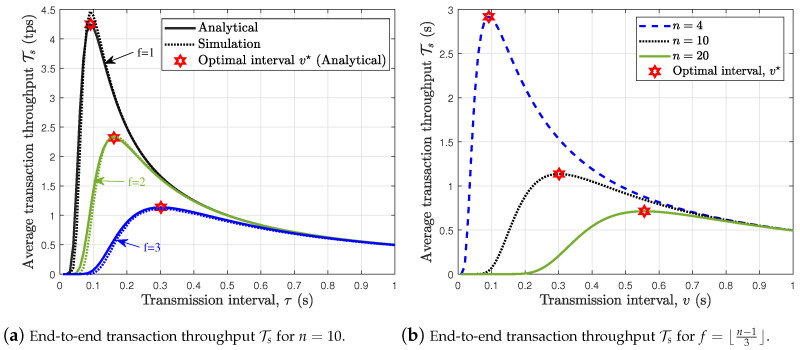
End-to-end transaction throughput of wireless PBFT network.

In Figure 6a,b, we plot the average end-to-end transaction throughput against the transmission interval for n=10 (f=1,2,3) and n=10,20,50 ($f=\lfloor \frac{n-1}{3}\rfloor$), respectively. Results show that there exists an optimal transmission interval that maximizes the average end-to-end transaction throughput. Figure 6a further shows that increasing *f* increases the optimal transmission interval $v^{★}$ while reducing the optimal end-to-end transaction throughput. In addition, Figure 6b shows that a larger PBFT network such as the case with n=20 requires a much larger transmission interval than the case with n=4 in order to achieve the optimal end-to-end transaction throughput $T_{s}\left(v^{★}\right)$. The parameters $v^{★}$ and $T_{s}\left(v^{★}\right)$ can serve as input when setting the PBFT network’s transaction timer. Note that at the expiration of the timer without a reply from the nodes, the client resends the service request to the primary node of the PBFT network.

**Figure 7:**
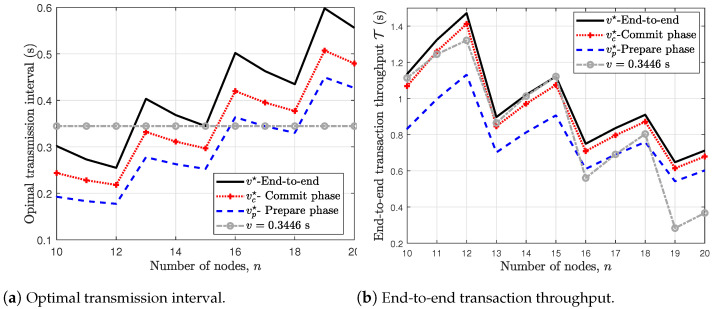
Effect of increasing the number of node *n* for $f=\lfloor \frac{n-1}{3}\rfloor$.

In Figure 7a,b, we show the effect of increasing the number of nodes *n* on the optimal transmission interval and the end-to-end transaction throughput, respectively, for $f=\lfloor \frac{n-1}{3}\rfloor$. The sawtooth shape of the curves of Figure 7a,b are due to the fact that $f=\lfloor \frac{n-1}{3}\rfloor$. At instances n=10,13,16,19, the corresponding *f* increases, i.e., f=3,4,5,6 and the optimal transmission interval increases as seen earlier in Figure 6. On the other hand, at instances n=10,11,12 which all corresponds to f=3, the optimal transmission interval reduces. This is due to the fact that the probability of a node receiving prepare or commit messages from non-faulty nodes increases and out-weighs the effect of the collision as a result of the additional non-faulty node.

In Figure 7a, we compare the optimal end-to-end transmission interval $v^{★}$ with the optimal intervals obtained for the independent commit and prepare phases, i.e. $v_{c}^{★}$ and $v_{p}^{★}$, respectively. It can be observed that the optimal end-to-end transmission interval $v^{★}$ exceeds the optimal values for independent prepare and commit phases. Further, the optimal transmission interval for the commit phase exceeds that for the prepare phase, i.e. $v_{c}^{★}>v_{p}^{★}$. This observation is due to the fact the number of contending nodes for channel access in the commit phase exceeds that of the prepare phase since the primary does not broadcast in the prepare phase but the pre-prepare and the commit phases. Results in Figure 7b shows that the end-to-end based approach results in much higher transaction throughput, i.e., more transactions can be added to the blockchain. Moreover, we also benchmark our result with the case with fixed transmission interval, i.e., v=0.3446s (which is the optimal transmission interval for the case with n=15). As can be seen in Figure 7b, using a fixed transmission interval is sub-optimal as it leads to much lower transaction throughput, and hence, demonstrates the need for the optimization of the transmission interval.

**Figure 8:**
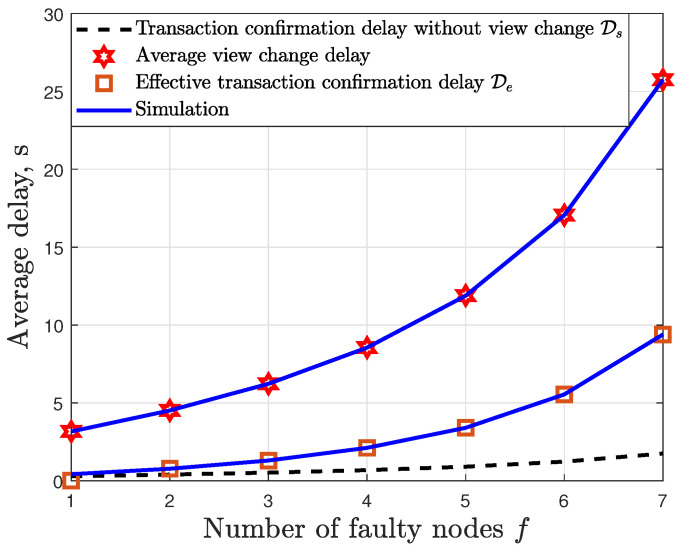
Effect of the view change on the PBFT confirmation delay.

In Figure 8, we show the results for the transaction confirmation delay when the effect of view change has been integrated. We plot the transaction confirmation delay without view change $D_{s}$, the average view change delay and the effective transaction confirmation delay $D_{e}$ for the case with $N=22,\zeta =10D_{s}$. The transaction confirmation delay without view change was obtained from (16). The results shows a tight match between the simulated and theoretical results. It can be seen that the view change leads to a significant increase in the effective transaction confirmation delay $D_{e}$. Furthermore, increasing the number of faulty nodes *f* also leads to an increase in the effective transaction confirmation delay.

**Figure 9:**
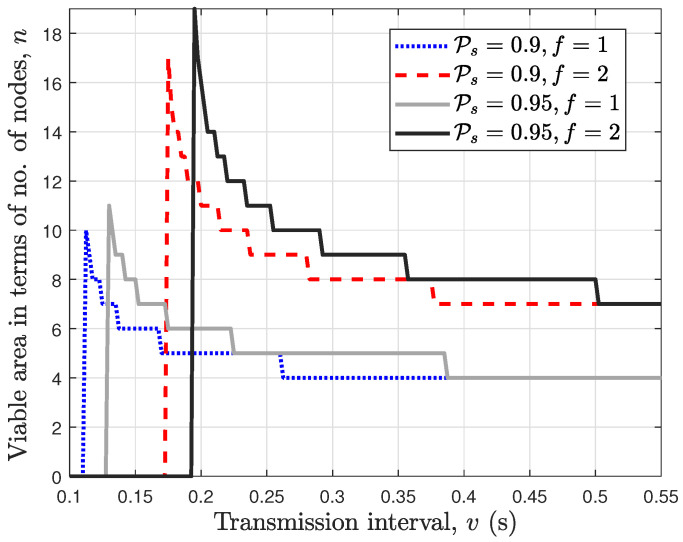
Viable area of PBFT consensus network expressed as a function of the number of nodes *n*.

In Figure 9, we plot the viable area of the wireless PBFT network against the transmission interval *v* for a fixed end-to-end success probability $P_{s}$ and number of faulty nodes *f*. For the case with $P_{s}=0.95$ and f=2, it can be seen that the wireless PBFT network becomes unviable for a transmission interval v<0.19s where n=0. Moreover, this constraint can be relaxed by either setting a more relaxed end-to-end success probability requirement as in the case with $P_{s}=0.9,f=2$ or reducing the number of faulty nodes as in the case with $P_{s}=0.95,f=1$. In other words, a much lower transmission interval *v* is required for the wireless PBFT network to become viable when either the end-to-end success probability or the number of faulty nodes is lowered. Further, for the case with $P_{s}=0.95$ and f=2, it can be observed that once the network becomes viable, the viable area and equivalently the number of nodes *n* reduces as the transmission interval *v* increases. Moreover, the viable area converges to the effective area of the minimum number of nodes required to achieve *constraint 1*, i.e., n=3f+1 with a further increase in the transmission interval *v*.

## 6. Conclusions

In this paper, we investigated the performance of the PBFT protocol when implemented over the wireless network. We first introduced the wireless PBFT network system model and the channel contention assumptions. The success probabilities for the prepare and commit phases, and the end-to-end success probability have been derived for the wireless PBFT network. Using the end-to-end success probability as the basis, we derived the expression for other KPIs of the wireless PBFT network namely, the transaction throughput, transaction confirmation delay, the optimal transmission interval, the average node transmit power and the viable area. We also derived the effective transaction confirmation delay to show the effect of the view change on the wireless PBFT networks. Numerical results validated the accuracy of our theoretical analysis. Furthermore, the viable area of the wireless PBFT network achieves liveness, resilience, and safety with the minimum number of nodes, minimum transmission interval, and minimum broadcast transmit power and thus results in significant energy savings.

Note that the analysis of the wireless PBFT network presented in this paper was based on the static IoT network scenario. Since most IoT devices intrinsically work over mobile systems or evolve toward mobility, performance analysis of the wireless PBFT networks that capture mobility deserves attention in future studies. In addition, the analysis only considers the collisions that might affect the system’s effectiveness and efficiency of the consensus. Therefore, the impacts of malicious attacks on the security of the system are also worth investigating in future studies. Moreover, the implementation of other wireless protocols for the PBFT network is also worth exploring.

## Data Availability

The data presented in this study are available on request from the corresponding author.
